# Supplementary material for: A Critical View of the Application of the APEX Software (Aqueous Photochemistry of Environmentally-Occurring Xenobiotics) to Predict Photoreaction Kinetics in Surface Freshwaters
Source: Molecules. 2019 Dec 18;25(1):9. doi: 10.3390/molecules25010009 (PMC7017383; doi:10.3390/molecules25010009)
Supplement: Supplementary file 1 [file molecules-25-00009-s001.zip › 3.2.4_gcc-4.4.0.zip!/3.2.4_gcc-4.4.0/3.2.4_gcc-4.4.0/doc/octave/html/interpreter/Calling-Other-Functions-in-Mex_002dFiles.html]

Calling Other Functions in Mex-Files - Untitled


Previous: Sparse Matrices with Mex-Files,
Up: Mex-Files


---

#### A.2.7 Calling Other Functions in Mex-Files

It is also possible call other Octave functions from within a mex-file
using `mexCallMATLAB`. An example of the use of
`mexCallMATLAB` can be see in the example below

```
```
     #include "mex.h"
     
     void
     mexFunction (int nlhs, mxArray* plhs[], int nrhs, 
     	     const mxArray* prhs[])
     {
       char *str;
     
       mexPrintf ("Hello, World!\n");
     
       mexPrintf ("I have %d inputs and %d outputs\n", nrhs,
     	     nlhs);
     
       if (nrhs < 1 || ! mxIsString (prhs[0])) 
         mexErrMsgTxt ("function name expected");
     
       str = mxArrayToString (prhs[0]);
     
       mexPrintf ("I'm going to call the function %s\n", str);
     
       mexCallMATLAB (nlhs, plhs, nrhs-1, prhs+1, str);
     
       mxFree (str);
     }
```
```

If this code is in the file myfeval.c, and is compiled to
myfeval.mex, then an example of its use is

```
     myfeval("sin", 1)
     a = myfeval("sin", 1)
     => Hello, World!
         I have 2 inputs and 1 outputs
         I'm going to call the interpreter function sin
         a =  0.84147
```

Note that it is not possible to use function handles or inline functions
within a mex-file.
